# Intuitive decision-making promotes rewarding prosocial others independent of the personality trait Honesty-Humility

**DOI:** 10.1038/s41598-020-75255-7

**Published:** 2020-10-29

**Authors:** Laila Nockur, Stefan Pfattheicher

**Affiliations:** 1 Department of Social Psychology, Ulm University, Albert-Einstein-Allee 47, 89077 Ulm, Germany; 2 Department of Psychology and Behavioural Sciences, Aarhus University, Bartholins Allé 11, 8000 Aarhus C, Denmark

**Keywords:** Psychology, Human behaviour

## Abstract

Although past research has convincingly shown that rewarding prosocial individuals helps to establish high levels of cooperation, research investigating factors that promote rewarding others has been surprisingly rare. The present research addresses this gap and examines two factors that were shown in past research to play a role in prosocial behaviour. In a well-powered study (total *N* = 1003), we tested the impact of (a) a basic prosocial personality trait (the Honesty-Humility dimension from the HEXACO personality model) and (b) intuitive decision-making, as well as (c) their interaction, in rewarding prosocial individuals. We found that (1) intuition promotes rewarding prosocial others; (2) Honesty-Humility was not significantly related to rewarding prosocial others; and (3) that Honesty-Humility did not significantly moderate the effect of intuition on reward. Implications for the understanding of reciprocating others’ prosocial behaviour are discussed.

## Introduction

Reward and punishment are prominent mechanisms to foster socially desired behaviour in human societies. When reward or punishment are executed as individual reactions to the actions of another individual, rewarding or punishing that individual constitutes reciprocal behaviour. Prosocial behaviour—behaviour that benefits others—is often reciprocated with reward (i.e., positive reciprocity) whereas antisocial behaviour—behaviour that harms others—is often reciprocated with punishment (i.e., negative reciprocity)^1^. The effectiveness of reciprocating previous behaviour has been shown in the context of cooperation, in which social welfare can be maximized by the joint contributions of the individuals involved: Rewarding cooperative and punishing uncooperative others, has been shown to increase cooperative behaviour—even among anonymous strangers—to a remarkable extent^2^. In fact, reward and punishment help to establish high levels of cooperation in situations where individuals actually have incentives to refrain from cooperation^2, 3^. Reciprocating behaviour (i.e., punishing uncooperative and rewarding cooperative individuals) is even observed when it implies personal costs^4–6^. What is more, individuals also engage in indirect reciprocity by rewarding individuals who showed prosocial behaviour towards a third person^7, 8^.

Whereas past research has revealed deep insights in the motivational and cognitive underpinnings of punishing uncooperative individuals (for a review, see^9^), research investigating factors that promote rewarding prosocial or cooperative others has been surprisingly rare. The present investigation addresses this gap and examines two factors that were shown in past research to play a role in prosocial behaviour. Specifically, the present research tests the impact of (a) a basic prosocial personality dimension (i.e., the Honesty-Humility dimension from the HEXACO personality model) and (b) intuitive decision-making, as well as (c) their interaction, in rewarding individuals (i.e., third-party reward).

Overall, the present investigation advances the existing literature in several ways. First, to our knowledge the present work is the first that examines the potential impact of personality traits and decision-making style in rewarding prosocial others. As such, the present contribution has the potential to create a better understanding about the dispositional and cognitive underpinnings of rewarding prosociality. Second, past research on intuitive decision-making in cooperative situations has led to a lively debate about whether cooperation is typically driven by intuitive and/or reflective processing (for a recent review, see^10^). The impact of intuitive decision-making has also been tested in the contexts of cheating^11–13^, and punishment of uncooperative individuals^14, 15^, as well as punishment of cooperative individuals^9^. With the present investigation, we integrate into the debate the important but largely neglected case of rewarding prosocial others. Third, past research has shown that Honesty-Humility promotes prosocial behaviour across a vast variety of different behaviours and across differently structured social situations^16–21^. We extend this research and test whether Honesty-Humility promotes rewarding (prosocial) others, and whether individuals high in Honesty-Humility reward others when asked to decide intuitively.

The following sections give an overview about the two factors examined in the present study (Honesty-Humility; intuitive decision-making). Subsequently, we outline why Honesty-Humility and intuitive decision-making might promote rewarding prosocial others.

Honesty-Humility is one factor of the HEXACO personality model^22^. The HEXACO model comprises the six factors of Honesty-Humility (H), Emotionality (E), Extraversion, (X), Agreeableness (A), Conscientiousness (C), and Openness to experience (O). Ashton and colleagues provide a detailed discussion regarding the differences of Neuroticism versus Emotionality and Agreeableness as conceptualised in traditional five-factor models of personality and the HEXACO model^23^. According to Ashton and Lee (p. 156), “Honesty-Humility represents the tendency to be fair and genuine in dealing with others, in the sense of cooperating with others even when one might exploit them without suffering retaliation.” In this regard, individuals high in Honesty-Humility are described to be sincere, fair, modest, and greed avoidant^22^.

Past empirical research has convincingly shown the positive impact of Honesty-Humility on a vast variety of prosocial behaviour^21, 24, 25^. It has been documented that Honesty-Humility is positively related to sharing money with an anonymous partner^16–18, 20, 26, 27^, trustworthiness^28^, trusting others^29^, interpersonal and intergroup cooperation^30–32^, reduced workplace delinquency^33, 34^, and reduced cheating behaviour^35–38^.

To sum up, there is convincing evidence for Honesty-Humility to promote prosocial behaviour. In the present contribution, we examine whether we can extend this line of research, in that we examine the relation of Honesty-Humility and rewarding (prosocial) others. This relation does not seem to be obvious, and in fact, it might not appear at all. On the one hand, Honesty-Humility is related to prosocial behaviour, and arguably, rewarding prosocial others is a prosocial action. It has already been shown that non-exploitation as one core characteristic of Honesty-Humility is linked to positive reciprocity, that is responding with kindness to others’ kind behaviour^1^. In addition, individuals high in Honesty-Humility may want to establish a prosocial environment in general, which might also foster rewarding prosocial others. From that perspective, one should expect that individuals high in Honesty-Humility reward prosocial others.

On the other hand, rewarding prosocial others does not closely fit the essence of Honesty-Humility: Rewarding prosocial others is *reactive* behaviour (i.e., prosocial behaviour as a reaction to behaviour shown by another individual), whereas Honesty-Humility unfolds its prosocial impact especially in proactive prosocial situations, for instance when one has to decide whether or not to cooperate^16, 30^. From the latter perspective, it does not follow that Honesty-Humility relates to rewarding prosocial others. In the end, it is an empirical question whether Honesty-Humility relates to rewarding prosocial others. The study presented below puts the relation to an empirical test. In the next section, we explore the impact of intuitive decision-making on prosocial behaviour.

In a series of experiments, Rand et al. document that conditions fostering intuitive decision-making (e.g., priming of an intuitive decision-making style) increase prosocial behaviour in the group context (e.g., cooperative behaviour in public good games), whereas conditions that inhibit intuitive decision-making (e.g., a time delay prior to decisions) decrease cooperation^39–41^. A direct replication^42^, a registered replication report^43^ (for a response, see^44^), and a meta-analysis^45^ have found contradictory results, with the effect depending on whether participants who do not comply with the instructions are excluded or not^42, 43^ and the manipulation used^45, 46^. Rand’s recent updated meta-analysis^46^, however, supports the positive link between intuition and cooperation.

To sum up, there is mixed evidence for a main effect of intuition on cooperation. The theoretical notion that cooperation should emerge intuitively is grounded in the Social Heuristics Hypothesis (SHH^39, 47, 48^). The SHH postulates that individuals who learned and experienced that cooperation reflects a beneficial strategy in daily life should apply this strategy per default (i.e., intuitively) in new and atypical situations, for instance, in the new situation of playing a public goods game in the laboratory. In contrast, individuals who did not learn and experience that cooperation reflects a beneficial strategy in daily life should possess an uncooperative default mode and therefore should not cooperate intuitively in new and atypical situations. In line with the SHH, cooperation is only intuitive for those individuals who learned and experienced that cooperation reflects a beneficial strategy^39, 40, 49–52^. It seems that basic social preferences are revealed when individuals are forced to make decisions intuitively^9, 49^.

On basis of the SHH, one can deduce two hypotheses for the case of rewarding prosocial others. First, based on the assumption that individuals learned and experienced that rewarding others is a beneficial strategy in daily life, because it is a pleasurable experience^53^, and reciprocally responded^7, 54–56^, one can expect that individuals execute rewarding prosocial others as automatic intuition. Building on the assumption that it is individuals high in Honesty-Humility who have experienced and learned that prosocial behaviour can be beneficial^22, 23^, individuals high in Honesty-Humility should, according to the SHH, execute their basic prosocial tendencies as automatic intuitions. On this basis, one can assume that individuals high in Honesty-Humility reward prosocial others intuitively. Second, flipped predictions can be made for individuals low in Honesty-Humility. On the basis that individuals low in Honesty-Humility have experienced and learned that uncooperative, egoistic behaviour is beneficial for them, individuals low in Honesty-Humility should, according to the SHH, execute their antisocial tendencies as automatic intuitions. In line with these ideas, individuals low in Honesty-Humility should show a decrease in rewarding others’ prosocial behaviour when asked to decide intuitively.

In sum, we studied several ideas in the investigation reported below. First, we tested whether Honesty-Humility is positively associated with rewarding prosocial others. Second, we examined whether intuition promotes rewarding prosocial others. Third, we tested whether this effect depends on individuals’ level of Honesty-Humility, in that individuals high in Honesty-Humility increase in rewarding behaviour when asked to decide intuitively, and individuals low in Honesty-Humility decrease in rewarding behaviour when asked to decide intuitively.

In the present study, participants were presented the decision that another participant in a previous study had made as the allocator in a dictator game. In the dictator game, the allocator was endowed with a certain amount of money and could decide whether to keep all of it or to share it equally with another participant. Participants in the present study saw the decision of one allocator (i.e., the allocator kept all money or shared it equally) and could then reward the allocator. Before this third-party reward task, participants filled out items to assess the HEXACO personality dimensions. For exploratory purposes, we also assessed participants’ Social Value Orientation (SVO)^57^. We experimentally manipulated decision-making style (intuition vs. reflection vs. a control condition) when deciding how much to give to the allocator as a reward (see “Methods” section for more details on the experimental procedure).

## Results

**Figure 1:**
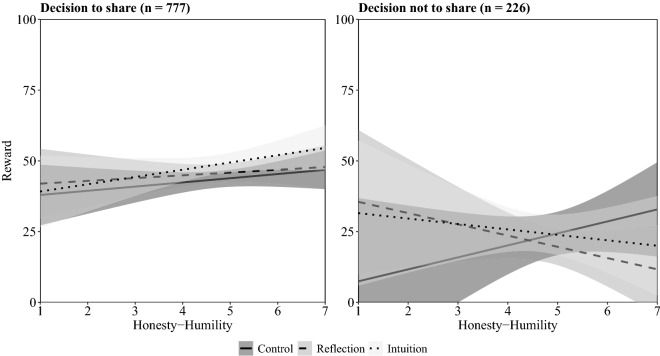
Reward depending on decision of the allocator (to share or not to share), decision-making style, and Honesty-Humility. The full range of all scales is displayed; shaded areas represent 95% confidence intervals.

Prosocial allocators (*n* = 777), that is allocators who chose to share the money with the other participant, were on average rewarded with 45.89 of 100 money units (*SD* = 25.90). We used generalized linear regression models to explore the association between decision-making style and Honesty-Humility with reward behaviour. As displayed in Table 1, the reward amount was significantly increased by intuitive decision-making (*M* = 48.87, *SD* = 26.38) but not by reflective decision-making (*M* = 45.67, *SD* = 26.56) compared to the control condition (*M* = 43.51, *SD* = 24.62; see Table 1, Model 1). This effect remained robust when the HEXACO dimensions, Social Value Orientation, as well as demographic variables were included in the analysis (see Table S5 in the Supplementary Information). Reward behaviour did not differ significantly between the intuitive and reflective decision-making conditions. Honesty-Humility was positively but not significantly associated with reward amount (see Table 1, Model 2; see Table S1 in the Supplementary Information for zero-order correlations between Honesty-Humility and reward behaviour across and within experimental conditions). We did not find evidence that the effect of intuitive or reflective decision-making depended on Honesty-Humility (see Table 1, Model 3, and Fig. 1, left panel). As such, we did not find evidence for the idea that individuals high in Honesty-Humility reward prosocial others intuitively while individuals low in Honesty-Humility execute their antisocial tendencies as automatic intuitions.

**Table 1:** Results of the regression analyses to predict rewarding of prosocial allocators (*N* = 777).

	Estimate	95% CI	SE	β	p
**Model 1 (R**^***2***^** = 0.01)**					
Intercept	43.52	40.44, 46.59	1.57		< 0.001
Intuition	5.35	0.85, 9.85	2.29	0.10	0.020
Reflection	2.15	− 2.22, 6.52	2.23	0.04	0.334
**Model 2 (R**^***2***^** = 0.01)**					
Intercept	45.89	44.07, 47.72	0.93		< 0.001
H	1.66	− 0.09, 3.40	0.89	0.07	0.062
**Model 3 (R**^***2***^** = 0.01)**					
Intercept	43.54	40.46, 46.61	1.57		< 0.001
H	1.48	− 1.39, 4.35	1.46	0.06	0.311
Intuition	5.32	0.82, 9.82	2.29	0.09	0.021
Reflection	2.12	− 2.25, 6.48	2.23	0.04	0.342
H × Intuition	1.09	− 3.19, 5.36	2.18	0.02	0.618
H × Reflection	− 0.50	− 4.68, 3.68	2.13	− 0.01	0.814

Intuition and Reflection are dummy coded with control as reference.

*H* Honesty-Humility; H is mean-centred.

Similar results were found when including Social Value Orientation (SVO) as basic prosocial disposition instead of Honesty-Humility: Independent of decision-making style, a more prosocial SVO predicted higher amounts of reward for prosocial allocators (see Tables S2 and S5 in the Supplementary Information). The association between Honesty-Humility and reward behaviour was smaller and non-significant when SVO was included in the model, whereas the effect of intuition did not change when SVO was included (see Table S5 in the Supplementary Information). None of the other HEXACO dimensions was associated with rewarding a prosocial allocator (see Tables S1 and S5 in the Supplementary Information).

Allocators who chose *not* to share the endowment (*n* = 226) were rewarded with significantly lower amounts (*M* = 23.03, *SD* = 29.72) than allocators who chose to share (*M* = 45.89, *SD* = 25.90); *b* = 22.87, *t*(1001) = -11.29, *p* < 0.001. This result remained robust when decision-making style, HEXACO dimensions, SVO and demographic variables were included in the model (see Table S7 in the Supplementary Information). The decision to reward an allocator who chose not to share was not explained by decision-making style (Intuition: *b* = 0.45, *p* = 0.927; Reflection: *b* = − 3.67, *p* = 0.460) nor Honesty-Humility (*b* = − 0.61, *p* = 0.754; see Table S4 in the Supplementary Information). A higher SVO angle (i.e., more prosociality) predicted lower amounts of reward for allocators who chose not to share only when individuals were asked to decide intuitively (see Table S3 in the Supplementary Information). Correlations between rewarding an allocator who chose not to share and the other HEXACO dimensions are reported in Table S1 in the Supplementary Information.

Summing up the most important findings, the results show that intuitive decision-making (compared to the control condition) promotes rewarding a prosocial individual—even if rewarding implies lower personal benefits. Individuals who were asked to think deliberatively about their decision did not differ from individuals in the control condition regarding the reward amount. We also did not find evidence for a significant association between Honesty-Humility and rewarding prosocial others, although the effect scratched at conventional levels of significance (the main effect’s p-value was *p* = 0.062).

## Discussion

Rewarding prosocial others is one important and efficient means to establish cooperation on high levels^2^. The present research has examined the association of rewarding (prosocial) others with two factors that were shown in past research to play a role in the context of prosocial behaviour—the personality dimension Honesty-Humility^17–21^ and intuitive decision-making^40, 58^. Several results were obtained in the present study. First, we show that being instructed to decide intuitively compared to receiving no instructions promotes rewarding prosocial others. Second, our data did not support the idea that Honesty-Humility is significantly (at conventional levels of *p* < 0.05) related to rewarding prosocial others. Third, we also found no significant evidence for the idea that intuition promotes or reduces rewarding prosocial others dependent on individual differences in Honesty-Humility. These results have implications for research on intuitive decision-making in the context of prosocial behaviour and on Honesty-Humility. We discuss these implications in the following sections.

Past research has revealed mixed evidence for an intuitive cooperation (main) effect, ranging from failed direct replications^42^, a failed replication in a large registered replication report^43^, but see^44^, and a meta-analysis showing contradictory results^45^ to a recent updated meta-analysis^46^ which finds supports the positive link between intuition and cooperation. The present research supports the basic notion that intuition can promote prosocial behaviour—specifically, rewarding prosocial others. As such, we can add to previous literature showing the effect of intuition on different forms of social behaviour (e.g., honesty and cheating^11^; helping^59^; punishment^9, 15^). Although the effect size of the intuition effect seems rather small, an increase in reward of more than five percentage points is comparable to the association between intuition and cooperation found in meta-analyses^45–47^. It should be noted that we merely instructed participants to decide intuitively or deliberatively, and we do not know how many participants complied to this instruction. Thus, the effect observed in our study represents the intent-to-treat effect which might underestimate the true effect^46^. Of note, one strength of the present study is that we contrasted intuitive decision-making against a control condition (instead of a reflective condition like most other research^46^), which allows us to disentangle the effects of intuitive and reflective decision-making.

Having shown an effect of intuition on rewarding prosocial others, the question is for whom does this effect more likely emerge. Based on the SHH, we tested the idea that individuals high in Honesty-Humility express their prosocial tendencies as automatic intuitions and reward prosocial others intuitively. We did not find empirical support for this idea. It could be that rewarding prosocial others does not fit the essence of Honesty-Humility, because rewarding prosocial others reflects reactive prosocial behaviour, whereas Honesty-Humility reveals its prosocial impact especially when it comes to proactive prosocial behaviour^16, 30^. In fact, overall, we only observed a weak and non-significant association between Honesty-Humility and rewarding of prosocial individuals. Although there is evidence that non-exploitation as a core feature of Honesty-Humility is associated with positive reciprocity^1^, we did not find support for the idea that individuals high in Honesty-Humility also engage in indirect reciprocity by rewarding prosocial others in a third-party reward task. Given the p-value of the main effect of Honesty-Humility close to *p* = 0.05, future research needs to test whether there is a (small) main effect of Honesty-Humility on rewarding prosocial others, or whether the effect is indeed rather close to zero. In fact, because personality dimensions are considered stable and therefore expected to affect behaviour in many situations, even a small association between Honesty-Humility and rewarding prosocial others could have a substantial impact on the social environment of individuals over time^60^.

Still, the findings of the present study also speak to the possibility that rewarding prosocial others cannot simply be added to the long list of prosocial behaviours that were found to be substantially related to Honesty-Humility^34, 61, 62^. The lack of a noteworthy relation between Honesty-Humility and rewarding prosocial others, as shown in the present study, is important because it provides information about cases where Honesty-Humility might not relate to prosocial behaviour (see also^29^). In this way, the present contribution may inspire other research investigating under what conditions Honesty-Humility is or is *not* related to prosocial behaviour. Showing conditions under which Honesty-Humility is not related to prosocial behaviour or even related to harmful behaviour (e.g., when prosocial behaviour towards the ingroup harms the outgroup^63^, but see^32^) seems to be an important next step in research on Honesty-Humility, so that a more differentiated picture could be drawn about this important prosocial disposition.

Finally, we want to acknowledge limitations of the present investigation and point to potential future research. First, past research has shown that the effect of intuition on cooperation depends on the manipulation used: meta-analytic evidence suggests that the induction method used to manipulate decision-making style in the present investigation yields larger effects on prosocial behaviour compared to other methods (e.g., time constraints)^45, 47^. In this regard, it is unclear whether the findings of the present study generalize across different manipulations of intuitive decision-making. Still, we argue that does not undermine the findings of the present study per se; it is that the mere generalizability is so far unclear.

Second, it is a question for future research whether the present research findings generalize to different operationalizations of reward and to different modalities. For instance, if reward is made more costly or the money that could be invested in rewarding the allocator was earned instead of windfall profit, possibly only individuals with true prosocial preferences (i.e., individuals high in Honesty-Humility) would reward prosocial others. In this (hypothetical) case, a positive relation between Honesty-Humility and rewarding prosocial others might emerge. Also, when changing to different modalities of rewarding prosocial others (e.g., praise), it is not clear whether the found (non-)relations of the present study would hold. Thus, the present findings apply only to third-party reward; it is an open question whether the effects generalize across different games, operationalizations of reward behaviour, and modalities, which also includes reward behaviour in applied settings in real life.

Third, the present investigation has looked at reward only on a short time scale. In order to better understand the evolution of cooperation, one actually must take into account long-term consequences and dynamics (as modelled, for instance, in^64^). Higher levels of reward fostered by intuition might pay out in the long run because rewarded individuals keep up their prosocial behaviour. In this way, intuitive rewarding might be an adaptive strategy that is beneficial. This, however, must still be shown.

To conclude, the present investigation provides a better understanding of an important factor shown to promote cooperation—rewarding prosocial others. Given the lack of previous psychological research on rewarding prosocial others, the present work can be considered an initial step and might open a new avenue of research for studying the motivations and cognitions of rewarding (prosocial) others.

## Methods

### Research ethics statement

The present investigation was conducted in full accordance with the ethical guidelines of the American Psychological Association (APA). The manipulation of decision-making style to study its effect on rewarding prosocial others was approved by the central Research Ethics Committee of Ulm University (application number 297/16). There was no deception of participants. All participants gave informed consent before starting the study protocol, and the study was conducted in accordance with relevant guidelines and regulations.

### Procedure

Participants first responded to the HEXACO-60 and the SVO slider measure (see details below). Then they were informed that in a previous study, participants were randomly assigned the role of an “allocator” or a “recipient” in a game involving money (i.e., the “dictator game”). The allocator in the previous study was endowed with 100 monetary units (MU) and could decide whether to share it equally with the recipient or to keep all of it. Participants were informed that only the allocator had control over the final outcome; the recipient had to accept the decision by the allocator. In their instructions, participants in the present study were told that they would see the decision that one of the allocators actually made. Participants were then endowed with 100 MU and could decide how many they wanted to give to the allocator from the previous study as a reward. Their own bonus payment would be reduced by whatever they decided to give away to the allocator from the previous study. One hundred MU in the experiment equalled $0.10. There was no deception in this study. Therefore, participants received the endowment of 100 MU minus the MU invested to reward the allocator as a bonus payment. The amount each participant invested in reward was paid to one randomly matched allocator from the previous study. The instructions used are provided on the Open Science Framework (OSF; https://osf.io/fjk24/). In order to proceed with the study, participants had to answer two questions ensuring their correct understanding of the instructions. Before being informed about the allocator’s choice and making the decision whether or not to reward him or her, participants were randomly assigned to one of three decision-making conditions.

### Manipulation of decision-making style

In line with Rand et al.^40, 46, 47^, we experimentally manipulated decision-making style when participants decided over the reward by either asking participants to decide intuitively (“We ask you to make your decision from the gut. That is, rely on your intuition and just follow your predominant feelings.”, intuition condition, *n* = 345) or asking them to decide deliberatively (“We ask you to think deliberatively about your decision. That is, consider pros and cons and reflect before you make your decision.”, reflection condition, *n* = 320). In this condition, participants could only proceed to the next page after 15 s, to give them time to think about their decision. In the control condition (*n* = 338), no such information was given. Participants then were presented the decision of a real allocator who shared the endowment (*n* = 777) or did not share the endowment (*n* = 226) and indicated how many monetary units they wanted to invest to reward the allocator (0–100).

### Measures

Participants filled out the 60-item version of the HEXACO Personality Inventory-Revised (HEXACO-60^65^; 10 items per dimension). The HEXACO assesses the basic personality dimensions Honesty-Humility (e.g., “I would never accept a bribe, even if it were very large.”), Emotionality (e.g., “I sometimes can’t help worrying about little things.”), Extraversion (e.g., “In social situations, I’m usually the one who makes the first move.”), Agreeableness (e.g., “Even when people make a lot of mistakes, I rarely say anything negative.”), Conscientiousness (e.g., “I often push myself very hard when trying to achieve a goal.”), and Openness (e.g., “I would enjoy creating a work of art, such as a novel, a song, or a painting.”) Participants responded on a scale ranging from 1 = “strongly disagree” to 7 = “strongly agree.” All six dimensions had adequate reliability (α > 0.76).

We also assessed participants’ Social Value Orientation (SVO) with the 6-item slider measure^57^. Participants had to decide how to allocate resources between themselves and a randomly matched other person over a defined continuum of joint payoffs. The task was incentivized as one of the decisions was randomly chosen and paid out to the participants. Of the participants, 68 were characterized as competitive, 312 as individualistic, 618 as prosocial, and 5 as altruistic. The SVO-angle ranged between − 58.00 and 61.39 (*M* = 23.27, *SD* = 19.67).

### Participants

We recruited 1000 participants through Amazon Mechanical Turk (MTurk^66^) to take part in a study about personality and decision-making. A total of 1115 participants agreed to take part in the study before the quota was fulfilled. One participant did not finish the survey. Another 111 were excluded because they failed to correctly answer one of two instructed response items (“This is an attention check. Please answer with ‘strongly agree’.”) The final sample therefore consisted of 1003 (606 female, 375 male, 22 n/a) US participants (*M*_age_ = 35.25, *SD* = 12.19). The number of participants was chosen to match the number of allocators from the previous study. To pay out the bonus payment according to the reward decisions, almost all participants were matched with exactly one allocator from the previous study (*N* = 999), while only four participants, who decided not to reward the allocator, were not actually matched but simply payed their bonus payment. For the main analyses, we only included the reward decision for allocators who decided to share the endowment (*N* = 777), since our hypotheses applied to this behaviour. A sensitivity analysis revealed that with this sample size, we can detect even small effects of *f*^2^ = 0.01 for single regression coefficients at an alpha level of 0.05 (two-tailed) with sufficient statistical power of 0.80 (the power analysis was done using G*Power^67^).

### Variables and statistical analysis

All analyses were conducted using linear regression analysis. To examine the effect of decision-making style, we used dummy variables for intuition (intuition = 1, reflection = 0, control = 0) and reflection (intuition = 0, reflection = 1, control = 0) with the control condition as reference group. To test the hypotheses, we first ran models including the focal variables (Honesty-Humility and decision-making style). To examine the robustness of the results, we also ran multivariate analyses in which we included the other HEXACO dimensions as well as Social Value Orientation and demographic variables. Results of these analyses are displayed in the “Supplementary Information”.

## Supplementary information

Supplementary Tables.

## Data Availability

All data files and instructions for the reward task are available on the OSF: https://osf.io/fjk24/.

## Code availability

The corresponding SPSS and R code is also available on the OSF: https://osf.io/fjk24/.
