# Supplementary material for: Intuitive decision-making promotes rewarding prosocial others independent of the personality trait Honesty-Humility
Source: Sci Rep. 2020 Oct 29;10:18579. doi: 10.1038/s41598-020-75255-7 (PMC7596041; doi:10.1038/s41598-020-75255-7)
Supplement: Supplementary file 1 — Supplementary Tables. [file 41598_2020_75255_MOESM1_ESM.pdf]

Supplementary Information

Intuitive decision making promotes rewarding prosocial others independent of the personality trait Honesty-Humility

Laila Nockur[1*], Stefan Pfattheicher[2]

[1] Ulm University, Germany; [2]Aarhus University, Denmark

Corresponding author:

Laila Nockur
Ulm University
Department of Social Psychology
Albert-Einstein-Allee 47
89077 Ulm, Germany
laila.nockur@uni-ulm.de

Table S1. Zero-order correlations with reward behaviour depending on decision of the allocator and decision-making style.

	Prosocial allocator (*n* = 777)				Proself allocator (*n* = 226)			
	Control	Intuitive	Reflective	Overall	Control	Intuitive	Reflective	Overall
H	.07	.10	.04	.07†	.15	-.06	-.14	-.02
E	-.01	.10	.02	.04	-.20	-.04	-.14	-.12†
X	.05	-.05	.05	.02	.22†	.20†	.11	.17**
A	-.01	.12†	.04	.05	.15	.02	.20†	.12†
C	-.02	-.01	-.07	-.03	.01	-.02	-.14	-.06
O	.02	-.03	.01	.00	.02	-.12	-.31**	-.14*
SVO	.19**	.17**	.20**	.19***	.03	-.34**	-.01	-.12†
Age	-.03	.002	-.07	-.04	.15	-.15	-.08	-.04
Female	-.03	.03	-.01	-.001	-.01	-.02	-.19	-.07

Note. † $p < .10$, * $p < .05$, ** $p < .01$, *** $p < .001$; H = Honesty-Humility, E = Emotionality, X = Extraversion, A = Agreeableness, C = Conscientiousness, O = Openness; SVO = Social Value Orientation (SVO angle).

Table S2. Results of the regression analyses to predict rewarding an allocator who decided to share by Social Value Orientation (SVO) and decision-making style.

		Estimate	*95% CI*	*SE*	*β*	*p*
Model 1	Intercept	45.76	43.97, 47.56	0.91		<.001
(R^2 = .03)	SVO	0.25	0.16, 0.34	0.05	.19	<.001
Model 2	Intercept	43.42	40.39, 46.45	1.54		<.001
(R^2 = .04)	SVO	0.25	0.09, 0.40	0.08	.18	.002
	Intuition	5.37	0.94, 9.80	2.26	.10	.018
	Reflection	2.02	-2.28, 6.32	2.19	.04	.357
	SVO x Intuition	-0.01	-0.24, 0.22	0.12	-.01	.900
	SVO x Reflection	0.02	-0.20, 0.24	0.11	.01	.865

Note. SVO = SVO angle; Intuition and Reflection are dummy coded with control as reference.

Table S3. Results of the regression analyses to predict rewarding an allocator who decided not to share by Social Value Orientation (SVO) and decision-making style.

		Estimate	*95% CI*	*SE*	*β*	*p*
Model 1	Intercept	22.72	18.83, 26.62	1.98		<.001
(R^2 = .01)	SVO	-0.17	-0.36, 0.02	0.10	-0.12	.084
Model 2	Intercept	24.15	17.03, 31.26	3.61		<.001
(R^2 = .03)	SVO	0.04	-0.29, 0.38	0.17	.03	.799
	Intuition	-0.73	-10.32, 8.87	4.87	-.01	.882
	Reflection	-3.75	-13.46, 5.97	4.93	-.06	.448
	SVO x Intuition	-0.56	-1.02, -0.09	0.24	-.22	.020
	SVO x Reflection	-0.06	-0.54, 0.41	0.24	-.02	.796

Note. SVO = SVO angle; Intuition and Reflection are dummy coded with control as reference.

Table S4. Results of the regression analyses to predict rewarding an allocator who decided not to share by Honesty-Humility and decision-making style.

		Estimate	*95% CI*	*SE*	*β*	*p*
Model 1	Intercept	23.03	19.12, 26.93	1.98		<.001
(R^2 < .01)	H	-0.61	-4.48, 3.25	1.96	-.02	.754
Model 2	Intercept	24.14	16.91, 31.36	3.67		<.001
(R^2 < .01)	Intuition	0.45	-9.26, 10.16	4.93	.01	.927
	Reflection	-3.69	-13.51, 6.13	4.98	-.06	.460
Model 3	Intercept	23.41	16.09, 30.73	3.71		<.001
(R^2 = .02)	H	4.23	-2.57, 11.04	3.45	.14	.222
	Intuition	0.89	-8.94, 10.72	4.99	.01	.859
	Reflection	-2.88	-12.76, 7.01	5.02	-.05	.567
	H x Intuition	-6.15	-15.67, 3.37	4.83	-.12	.204
	H x Reflection	-8.21	-17.83, 1.42	4.88	-.16	.094

Note. Intuition and Reflection are dummy coded with control as reference; H = Honesty-Humility; H is mean-centred.

Including decision-making style, all HEXACO dimensions, Social Value orientation (SVO), and demographic variables in the analysis revealed that intuitive decision making (vs. control) as well as a more prosocial Social Value Orientation (SVO) were associated with rewarding a prosocial allocator, while the association between Honesty-Humility and rewarding a prosocial allocator was not significant (see Table S5). None of these constructs predicted rewarding an allocator who decided not to share (see Table S6). Rewarding an allocator who decided not to share was negatively associated with Openness and positively associated with Extraversion (see Table S6).

Table S5. Results of the multivariate regression analyses to predict rewarding an allocator who decided to share by decision-making style, HEXACO variables, SVO, and demographic variables.

	Estimate	*95% CI*	*SE*	*β*	*p*
Intercept	47.66	40.82, 54.49	3.48		<.001
Intuition	4.83	0.38, 9.27	2.26	.09	.033
Reflection	1.63	-2.71, 5.97	2.21	.03	.461
H	1.03	-1.02, 3.09	1.05	.04	.324
E	1.79	-0.16, 3.74	0.99	.07	.071
X	0.76	-1.12, 2.65	0.96	.03	.426
A	0.78	-1.34, 2.89	1.08	.03	.471
C	-1.87	-3.99, 0.25	1.08	-.07	.084
O	-0.55	-2.34, 1.24	0.91	-.02	.546
SVO	0.24	0.15, 0.34	0.05	.18	<.001
Age	-0.07	-0.24, 0.09	0.08	-.03	.376
Female	-2.20	-6.35, 1.95	2.11	-.04	.298

Note. H = Honesty-Humility, E = Emotionality, X = Extraversion, A = Agreeableness, C = Conscientiousness, O = Openness; SVO = Social Value Orientation (SVO angle); Intuition and Reflection are dummy coded with control as reference; R^2 = .04.

Table S6. Results of the multivariate regression analyses to predict rewarding an allocator who decided not to share by decision-making style, HEXACO variables, SVO, and demographic variables.

	Estimate	*95% CI*	*SE*	*β*	*p*
Intercept	27.27	11.65, 42.90	7.93		<.001
Intuition	2.49	-7.32, 12.30	4.98	.04	.617
Reflection	-1.81	-11.70, 8.08	5.02	-.03	.718
H	1.37	-3.42, 6.16	2.43	.05	.572
E	-2.24	-6.53, 2.05	2.18	-.08	.304
X	5.22	0.74, 9.69	2.27	.17	.023
A	3.33	-1.63, 8.29	2.52	.10	.187
C	-2.62	-7.54, 2.30	2.50	-.08	.295
O	-4.19	-8.11, -0.27	1.99	-.15	.036
SVO	-0.12	-0.32, 0.09	0.10	-.08	.256
Age	-0.13	-0.50, 0.25	0.19	-.05	.512
Female	-1.58	-10.29, 7.14	4.42	-.03	.722

Note. H = Honesty-Humility, E = Emotionality, X = Extraversion, A = Agreeableness, C = Conscientiousness, O = Openness; SVO = Social Value Orientation (SVO angle); Intuition and Reflection are dummy coded with control as reference R^2 = .04.

Table S7. Results of the regression analyses to predict rewarding by the allcoator's decision, decision-making style, HEXACO variables, SVO, and demographic variables.

	Estimate	*95% CI*	*SE*	*β*	*p*
Intercept	26.52	19.41, 33.63	3.62		<.001
Allocator	22.70	18.74, 26.66	2.02	.34	<.001
Intuition	4.24	0.15, 8.33	2.08	.07	.042
Reflection	1.01	-3.01, 5.03	2.05	.02	.621
H	1.08	-0.84, 3.00	0.98	.04	.269
E	0.93	-0.87, 2.72	0.92	.03	.313
X	1.66	-0.10, 3.42	0.90	.06	.065
A	1.15	-0.83, 3.13	1.01	.04	.253
C	-1.90	-3.87, 0.06	1.00	-.06	.058
O	-1.23	-2.87, 0.42	0.84	-.05	.144
SVO	0.16	0.07, 0.24	0.05	.11	.001
Age	-0.11	-0.27, 0.04	0.08	-.05	.144
Female	-1.73	-5.52, 2.06	1.93	-.03	.371

Note. Allocator = decision to share the endowment (0 = decided not to share, 1 = decided to share); H = Honesty-Humility, E = Emotionality, X = Extraversion, A = Agreeableness, C = Conscientiousness, O = Openness; SVO = Social Value Orientation; SVO = SVO angle; Intuition and Reflection are dummy coded with control as reference; R^2 = .13.
